# Consumption of Energy Drinks and Their Effects on Sleep Quality among Students at the Copperbelt University School of Medicine in Zambia

**DOI:** 10.1155/2019/3434507

**Published:** 2019-02-03

**Authors:** Richard Kalima Mwape, David Mulenga

**Affiliations:** ^1^The Copperbelt University, Michael Chilufya Sata School of Medicine, P.O. Box 71191, Hilcrest, Ndola, Zambia; ^2^The Copperbelt University, Michael Chilufya Sata School of Medicine, Public Health Unit, P.O. Box 71191, Hilcrest, Ndola, Zambia

## Abstract

*Background*. Good sleep quality is cardinal to good health, and research has shown that it plays a fundamental role in immunity, learning, metabolism, and other biological functions. Energy drink consumption is a popular practice among college students in the United States. There has been little research done on the consumption of energy drinks and its effects in Zambian universities. The main objective was to determine the effects of energy drinks on sleep quality among students at the Copperbelt University School of Medicine. A self-administered questionnaire was administered to 195 undergraduate students at the Copperbelt University School of Medicine in their second- and third-year of study. Energy drink consumption and sleep quality were assessed by univariate, bivariate, and multivariate analyses. 157 students were enrolled into the study. The prevalence of energy drink consumption was 27.4% among participants. Studying for an exam was the most common reason for drinking energy drinks (83.7% of energy drink users). The majority of participants were found to be have poor sleep quality (59.6%). There was a statistically significant association between energy drink consumption and poor sleep quality (p value < 0.01). The findings of our study show that energy drink consumption is not a common practice in the Zambian university setting as the prevalence was 27.4%. Furthermore, the prevalence of poor sleep quality among Zambian university students is high and is significantly associated with energy drink consumption, and there is a need to better understand the patterns of energy drink use as well as their effects on various aspects of health including sleep quality in the Zambian setting. Further research should assess the knowledge of nonmedical students on the effects of energy drinks.

## 1. Introduction

According to the American Psychological Association (APA), sleep is one of the factors associated with a person's health. The recommended amount of sleep for an adult is 7-8 hours [1]. This is aided by a biological clock or circadian rhythm that is internal to humans and “cues” the body to sleep when it is time [2]. Good sleep quality is cardinal to good health; research has shown that it plays a fundamental role in immunity, learning, and memory [3, 4]. Poor sleep quality weakens the body's immune system and makes and an individual more susceptible to infections such as a cold or flu. On the other hand, good sleep quality has a positive effect on learning and memory, and it also helps regulate the body's metabolism [5]. Although sleep deprivation has not been shown to significantly affect a person's ability to perform physical tasks, studies have shown that it has an effect on cognitive and mental ability which may manifest as hallucinations or distorted perception [6].

Hershner and Chervin found that poor sleep quality can be caused by sleep disorders, use of technology before sleeping, consumption of alcohol, energy drinks, and use of stimulants. In addition, the consumption of energy drinks increases sleep latency and the effects of energy drinks can persist for up to 8 hours and can lead to somnolence. This can adversely affect a student's academics as irregular sleep patterns have been shown to have a negative impact on learning and memory [7].

Energy drinks contain caffeine, ginseng, taurine, guarana, sugar, and B vitamins; among these, caffeine is the major ingredient [8]. Caffeine, a methylxanthine is a well-known central nervous system (CNS) stimulant which causes increased alertness and deferred fatigue [9]. Caffeine also has effects on other body systems such as the cardiovascular system, where it increases the heart rate, peripheral resistance which leads to increased blood pressure, tachycardia, and palpitations and may also cause arrhythmias [10]. However, not all effects of caffeine are negative; its relaxing effects on bronchial smooth muscle are the rationale for the use of the related substance aminophylline in the treatment of bronchial asthma. According to Katzung and colleagues, caffeine is also a mild diuretic [9]. Long-term use of caffeine has consequences. It has been found that long-term caffeine consumers are at risk of dependency (so-called caffeine dependency syndrome) and develop withdrawal symptoms once off the caffeine [11].

According to Ibrahim and Iftikhar [12], energy drink consumption is a global public health problem. The use of these products comes with a myriad of health risks, ranging from palpitations [10] to arrhythmias [13], caffeine dependency syndrome [11], anxiety, [14] and irregular sleep patterns [15]. Their effects on sleep can adversely affect a student's academic life as somnolence and irregular sleep patterns have been shown to have a negative impact on learning and memory [7]. No study had been done to assess the effects of energy drinks on sleep quality among students at the Copperbelt University. This study focused on the effects of energy drinks on sleep quality among students at the Copperbelt University School of Medicine.

Among the various ingredients in energy drinks, caffeine has been found to be the major component responsible for the effects of energy drinks [16]. Caffeine, a methylxanthine, has cardiovascular, renal, metabolic, gastrointestinal, musculoskeletal, neurological, and psychological effects [15] and has been shown to reduce sleep quantity and quality and increase sleep latency [17–19]. Most of the literatures reviewed state that the effects of caffeine on sleep quality and quantity are quite significant [17–19].

Consumption of energy drinks is a worldwide public health problem particularly among adolescents and young adults [12]. A good number of studies have been done on the consumption of energy drinks among college students. Malinauskas and colleagues [16] studied energy drink consumption among college students in the United States and found that over 50% consumed over one energy drink per month with 67% citing inadequate sleep as the reason for taking energy drinks. Another study conducted by Lohsoonthorn and colleagues [20] in Thailand found that energy drink consumption had a positive correlation with poor sleep quality among college students. A similar study by Velez and colleagues [21] in Chile yielded similar conclusions.

Attila and Cakir [22] found that consumption of energy drinks was quite common among college students in Turkey and most of them were not aware of the effects or ingredients of energy drinks. However, the study did not explore the effects of energy drinks on sleep quality and only considered fourth year students for the research.

Not much has been published on energy drink consumption among college students in Africa. In one study, Buxton and Hagan [23] assessed the consumption of energy drinks among students in Ghana but only considered those who were athletes. Furthermore, their study did not explore the effects of energy drinks on sleep quality.

A study by Lemma and colleagues [24] in Ethiopia found that the consumption of energy drinks was associated with poor sleep quality. As much as 52.7% of the students had poor sleep quality. However, the poor sleep quality was not entirely due to energy drink consumption, as factors such as cigarette smoking and khat use were cited.

No literature was found on the consumption of energy drinks and their effects on sleep quality in Zambia. In light of this knowledge gap, the study was conducted with the general objective of determining the effects of energy drinks on sleep quality among students at the Copperbelt University's School of Medicine in Zambia. The specific objectives were

1. To determine the prevalence of energy drink consumption among students at the Copperbelt University School of Medicine.
2. To determine sleep quality in general among students at the Copperbelt University School of Medicine.
3. To determine the effect of energy drinks on sleep quality among students using the energy drinks at the Copperbelt University School of Medicine.

This study will raise awareness on the overall sleep quality of university students and the effects of energy drinks on sleep quality in the Zambian setting; furthermore it will give information that will help to evaluate whether students in Zambia read before embarking on use of certain beverages as the students that will be assessed are expected to have basic medical sciences knowledge. This information can then be used to develop appropriate interventions for universities in Zambia.

## 2. Materials and Methods

### 2.1. Study Design and Site

A cross-sectional quantitative study was done at the Copperbelt University School of Medicine on undergraduate students. The school is located in Hilcrest, in the city of Ndola, which is the provincial capital of the Copperbelt province in Zambia. The school is the first public medical school on the Copperbelt province and offers both undergraduate and postgraduate programs. The school has over 1000 undergraduate students. The undergraduate programs offered are Bachelor of Medicine and Surgery (MBChB), Bachelor of Dental Surgery (BDS), Bachelor of Clinical Medicine (BSc CM), and Bachelor of Science (BSc) in Biomedical Sciences.

### 2.2. Study Population and Sampling Procedure

The study population was every student enrolled in the School of Medicine at the Copperbelt University pursuing an undergraduate degree in their second- and third-year of study. Second- and third-year students are expected to have basic medical sciences knowledge as they are at that stage in their training (preclinical students). Nonprobability convenience sampling was used to select the participants.

### 2.3. Sample Size

Sample size was determined using the startcalc application in Epi Info version 7 with a population size of 700, Confidence level of 90%, confidence limits of 5% and expected prevalence of energy drink consumption which was assumed to be 50% based on a similar study done in Africa [23]. Sample size was calculated as 195.

### 2.4. Data Collection and Materials

#### 2.4.1. Procedure

Self-administered questionnaires were distributed to second- and third-year students at the Copperbelt University School of Medicine. No personal details that could be used to identify the participants were collected. The data collection was done during the period 11th June, 2018, to 26th June, 2018. The date collection was done in 15 days in order to capture students before the beginning of their vacation.

#### 2.4.2. Materials

A self-administered questionnaire was used that had three parts. Part A covered student details and part B covered energy drink consumption with the first question being a screening test for energy drink consumption as adapted from the research that was done by Malinauskas and colleagues [16]. Part C covered sleep quality using the Pittsburgh sleep quality index (PSQI) [25]. The PSQI has 7 subcomponents that are used to come up with a global PSQI score to assess sleep quality. The subcomponents are subjective sleep quality, sleep latency, sleep duration, habitual sleep efficiency, sleep disturbances, use of sleeping medication, and daytime dysfunction. A global PSQI score <6 was considered as good sleep quality whereas a score of 6 or more was considered as poor sleep quality [26].  Note S1 is in the supplementary materials for a copy of the questionnaire.

### 2.5. Data Analysis

After data was collected, it was entered and analyzed in SPSS version 23.

### 2.6. Ethical Consideration

Ethical approval was obtained from the ethics committee, Tropical Disease Research Center (TDRC). Permission to conduct the research was obtained from the Copperbelt University's School of Medicine. The participants were verbally given all the necessary information about the research and verbal consent was obtained before taking part in this research. Confidentiality was upheld. No information about the participants was exposed to anyone who was not part of the research team.

## 3. Results and Discussion

### 3.1. Results

#### 3.1.1. Characteristics of the Study Population

Out of the total 195 sample size, 157 participants were enrolled in the study. The average age of the participants included in the data analyses was 22.9 years. More than half of the participants (61.8%) were male and 61.4% were in their second year of study. Three-quarters were studying Bachelor of Medicine and Surgery (75.9%). In regard to energy drink consumption patterns among students, 27.4% of participants reported drinking more than one energy drink per month in an average month for the current academic year.  Table 1 summarizes the associations between sociodemographic characteristics and sleep quality according to PSQI. Of note, age, year of study, and energy drink consumption had a statistically significant association with quality of sleep.

#### 3.1.2. Pittsburgh Sleep Quality Index (PSQI)

Table 2shows the PSQI sleep components subscale distribution. Of note, the majority of participants were found to have poor sleep quality (59.6%). However, only 29.7% subjectively reported their sleep quality as fairly bad or very bad. Regarding sleep duration, 59.2% reported sleeping <5 hours per day, 26% reported longer sleep latency (>30 minutes), and 13.4% reported having daytime dysfunction. Approximately 29.1% were found to have poor sleep efficiency. There was no statistically significant association between gender and quality of sleep.

Table 3 shows the odds ratios for having poor sleep quality according to sex, energy drink consumption, and age. Adjusting for sex and age, energy drink users had higher odds of having poor sleep quality (OR = 13.54; 95% CI 3.67- 50.05) compared with nonusers; this association was statistically significant.

#### 3.1.3. Energy Drink Consumption Patterns

There were more male (79.1%) than female (20.9%) energy drink users (p = 0.006). Furthermore, the majority (85.7%) of energy drink users were second year students (p < 0.01). More than two-thirds (71.8%) of energy drink users were 21-24 years old and 17.9% were more than 24 years old, whereas 10.3% were 18-20 years old. Almost four-fifths (79.1%) consumed 1-4 energy drinks in an average month and 16.3% consumed 5-10 energy drinks in an average month whereas 4.7% consumed 11 or more energy drinks in an average month. There was no statistically significant association between the number of energy drinks consumed in an average month and the quality of sleep (p = 0.709)

*Types of Energy Drinks Consumed.* The energy drink Dragon was the most commonly used brand of energy drinks (34.9%), while 27.9% used Wild Cat energy drink, more than one-fifth (23.3%) used Kung Fu energy drink, and approximately ten percent (9.3%) used Red Bull energy drink, while 2.3% used Rage energy drink and 2.3% used Monster energy drink. There were no statistically significant association between the type of energy drink consumed and the quality of sleep (p = 0.743).

*Reasons for Energy Drink Use by Students.* Studying for an exam was the most common reason for drinking energy drinks (83.7% of energy drink users). Needing more energy was a reason for consuming energy drinks in the majority of energy drink users (67.4%). Approximately one-third (30.2%) drank while completing a course project, assignment, or report and 14% due to insufficient sleep, while 9.3% drank with alcohol while partying and 4.7% to treat a hangover. The energy drink consumption patterns for reasons reported by students are summarized in Table 4.

### 3.2. Discussion

The main purpose of this study was to determine energy drink consumption and their effects on sleep quality among undergraduate students at the Copperbelt University School of Medicine. The prevalence of energy drink consumption was low (27.4%) compared to the findings in similar studies [16, 20] where the prevalence was above 50%. This may be because the energy drink sector in Zambia has only recently begun to grow and a lot of people are not aware of its availability. Energy drink consumption was higher among men (79.1%) than women (20.9%), similar to the findings of another study [20] where the prevalence was 62.9% among men in a Thailand college. This may be because the advertisement of energy drinks mainly depicts young athletic men, and women seem to fear trying out new things. A study in the United States of America among undergraduate students found that masculinity and risk taking were associated with frequency of energy drink consumption [27]. Studying for an exam (84%) and needing more energy (67%) were common reasons for consuming energy drinks; this is similar to patterns observed in a similar study done in the United States of America among college students [16]. This suggests that students use energy drinks to enable them to cope with academic stress and to enable them to study and complete tasks.

There was a high prevalence of poor sleep quality (59.6%) among students. The prevalence of poor sleep quality was consistent with the results of similar studies done at tertiary institutions [20, 24]. Out of the 59.6% students with poor sleep quality score according to the PSQI, of note, only 29.7% subjectively reported having poor sleep quality. This indicates that students with poor sleep quality may be unaware of their poor quality of sleep or are not willing to acknowledge it and therefore they are unlikely to make changes in their sleep patterns. There was a higher frequency of poor sleep quality among males (42.7%) than females (32.7%). Similar studies found that the prevalence of poor sleep quality was similar for men and women [28] whereas others found that poor sleep quality was more among women than men [21, 29]; this is in contrast to the findings of this study. This may be due to cultural differences; Zambian boys are brought up to be husbands, fathers, and leaders while girls are brought up to be submissive wives and mothers. Consequently, the girls are overburdened compared to boys in the sharing of roles and chores in the household [30]. This may make the women better equipped at handling the independence that comes with being in university, whereas for men, being in university may be the first time that they have to live on their own and do chores and study and these extra responsibilities may affect their sleep quality more than their female counterparts.

Consumption of energy drinks was significantly associated with poor sleep quality. Of note, energy drink users had higher odds of having poor quality of sleep. This is consistent with other researches that have been done [20, 21]. Caffeine is a major ingredient in energy drinks [8] and believed to play a key role in the biological mechanism through which energy drinks affect sleep quality. Caffeine is a methylxanthine which acts as a central nervous system stimulant to increase alertness and defer fatigue, by acting as an adenosine receptor antagonist [9]. Adenosine plays a role in the regulation of sleep and wakefulness by inhibiting the release of excitatory neurotransmitters [20, 31]. Furthermore, another ingredient in energy drinks, namely, taurine, augments the effects of caffeine [32].

## 4. Conclusion

In summary, the findings of our study show that energy drink consumption is not a common practice in the Zambian university setting as the prevalence was 27.4%. Furthermore, the prevalence of poor sleep quality among Zambian university students is high and is significantly associated with energy drink consumption. This obviates a need for students to be aware of the effects of energy drinks on sleep quality. Poor sleep quality has been found to be associated with stress and poor academic performance among students [33]. Therefore, university students should know the importance of good sleep quality.

*Limitations.* This study did not assess other factors that can affect sleep quality such as alcohol and other stimulants. Moreover, it was not be able to assess the other effects of energy drinks on the students' health. Furthermore, it only assessed second- and third-year students. The cross-sectional self-administered nature of the study could have contributed to the degree of error due to the subjective nature of the assessment.

*Recommendations*

- Governments should educate the public on the daily recommended intake of caffeine, recommended age limits for consuming caffeine, and effects of caffeine on sleep quality and health in general.
- Further research is needed to assess the knowledge of nonmedical students on the effects of energy drinks on sleep quality as well as health in general.

## Figures and Tables

**Table 1:** Characteristics of the study population.

**Characteristics**		**All**	**Poor Sleep Quality**	**Good Sleep Quality**	**P-Value**
**Age (n = 132)**	18-20	13 (9.8%)	12 (15%)	1 (1.9%)	0.032
	21-24	101 (76.5%)	56 (70%)	45 (86.5%)	
	>24	18 (13.6%)	12 (15%)	6 (11.5%)	

**Gender (n=137)**	Male	82 (59.9%)	47 (56%)	35 (66%)	0.241
	Female	55 (40.1%)	37 (44%)	18 (34%)	

**Year of study (n = 137)**	Second year	84 (61.3%)	71 (86.6%)	13 (23.6%)	<0.01
	Third year	53 (38.7%)	11 (13.4%)	42 (76.4%)	

**Program (n = 118)**	MBChB	88 (74.6%)	56 (82.4%)	32 (64%)	0.047
	BDS	25 (21.2%)	11 (16.2%)	14 (28%)	
	BSc CM	5 (4.2%)	1 (1.5%)	4 (8%)	

**Use of Energy Drinks (n = 141)**	No	102 (27.7%)	49 (58.3%)	53 (93%)	<0.01
	Yes	39 (73.2%)	35 (41.7%)	4 (7%)	

**Table 2:** Pittsburgh Sleep Quality Index components by Gender.

**Characteristics**	**All**	**Females**	**Males**	**P value**
**Subjective sleep quality (n = 138)**				

(i) Very good	30 (21.7%)	14 (25.5%)	16 (19.3%)	0.353
(ii) Fairly good	67 (48.6%)	24 (43.6%)	43 (51.8%)	
(iii) Fairly bad	27 (19.6%)	9 (16.4%)	18 (21.7%)	
(iv) Very bad	14 (10.1%)	8 (14.5%)	6 (7.2%)	

**Sleep latency (n = 138)**				

(i) <15 minutes	65 (47.1%)	26 (47.3%)	39 (47.0%)	0.237
(ii) 16-30 minutes	47 (34.1%)	26 (27.3%)	21 (25.3%)	
(iii) 31-60 minutes	28 (20.3%)	8 (14.5%)	20 (24.1%)	
(iv) >60 minutes	9 (6.5%)	6 (10.9%)	3 (3.6%)	

**Sleep duration (n = 138)**				

(i) >7 hours	6 (4.3%)	2 (3.6%)	4 (4.8%)	0.495
(ii) 6-7 hours	14 (10.1%)	6 (10.9%)	8 (9.6%)	
(iii) 5-6 hours	36 (26.1%)	18 (32.7%)	18 (21.7%)	
(iv) <5 hours	82 (59.4%)	29 (52.7%)	53 (63.9%)	

**Sleep efficiency (n = 137)**				

(i) >85%	97 (70.8%)	38 (69.1%)	59 (72.0%)	0.584
(ii) 75-84%	19 (13.9%)	6 (10.9%)	13 (15.9%)	
(iii) 65-74%	6 (4.4%)	3 (5.5%)	3 (3.7%)	
(iv) <65%	15 (10.9%)	8 (14.5%)	7 (8.5%)	

**Sleep Disturbances (n = 137)**				

(i) Not during the past month	6 (4.4%)	2 (3.7%)	4 (4.8%)	0.072
(ii) Less than once a week	112 (81.8%)	40 (74.1%)	72 (86.7%)	
(iii) Once or twice a week	18 (13.1%)	12 (22.2%)	6 (7.2%)	
(iv) Three or more times a week	1 (0.7%)	0 (0.0%)	1 (1.2%)	

**Sleep Medication (n = 138)**				

(i) Not during the past month	127 (92.0%)	47 (85.5%)	80 (96.4%)	0.057
(ii) Less than once a week	10 (7.2%)	7 (12.7%)	3 (3.6%)	
(iii) Once or twice a week	1 (0.7%)	1 (1.8%)	0 (0.0%)	

**Daytime dysfunction (n = 138)**				

(i) Not a problem	64 (46.4%)	22 (40.0%)	42 (50.6%)	0.479
(ii) Only a slight problem	55 (39.9%)	23 (41.8%)	32 (38.6%)	
(iii) Somewhat of a problem	16 (11.6%)	8 (14.5%)	8 (9.6%)	
(iv) A very big problem	3 (2.2%)	2 (3.6%)	1 (1.2%)	

**Sleep Quality (n = 137)**				

(i) Good	84 (61.3%)	37 (67.3%)	47 (57.3%)	0.241
(ii) Poor	53 (38.7%)	18 (32.7%)	35 (42.7%)	

**Table 3:** Odds ratios for poor sleep quality according to PSQI.

**Characteristics**		**Bivariate Results**		**Multivariate Results**	
		P-value	Unadjusted OR (95% CI)	P-value	Adjusted OR (95% CI)
**Sex**	Male		1.00 (Reference)		1.00 (Reference)
	Female	0.24	1.53 (0.75-3.12)	0.09	2.082 (0.89-4.90)

**Energy drink consumption**	No		1.00 (Reference)		1.00 (Reference)
	Yes	< 0.001	9.46 (3.13-28.57)	<0.001	13.54 (3.67-50.05)

**Age**	18-20		1.00 (Reference)		1.00 (Reference)
	21-24	0.03	0.10 (0.01-0.83)	0.09	0.15 (0.02-1.34)
	>24	0.12	0.17 (0.02-1.60)	0.17	0.18 (0.02-2.01)

**Table 4:** Reasons given by students for taking energy drinks and the number of energy drinks taken (n = 43).

**Reason**	**Number of students**		**No. of Energy drinks ** **consumed**				**No. of times per month**					
			**1**		**2 or more**		**1-3**		**4-5**		**>5**	
	**(n)**	**(**%**)**	(n)	(%)	(n)	(%)	(n)	(%)	(n)	(%)	(n)	(%)
**Insufficient Sleep**	6	(14%)	6	(100%)	0	(0%)	2	(33.3%)	3	(50%)	1	(16.7%)

**Needing more energy**	29	(67.4%)	27	(93.1%)	2	(6.9%)	13	(44.8%)	9	(31%)	7	(24.1%)

**Studying for an exam**	36	(83.7%)	33	(91.7%)	3	(8.3%)	15	(41.7%)	13	(36.1%)	8	(22.2%)

**completing a course project, assignment or report**	13	(30.2%)	12	(92.3%)	1	(7.7%)	6	(46.2%)	5	(38.5%)	2	(15.4%)

**Drinking with alcohol while partying**	4	(9.3%)	4	(100%)	0	(0%)	2	(50%)	2	(50%)	0	(0%)

**Treating a hangover**	2	(4.7%)	2	(100%)	0	(0%)	1	(50%)	1	(50%)	0	(0%)

## Data Availability

The SPSS raw data used to support the findings of this study have been deposited in Mendeley Data Repository (https://dx.doi.org/10.17632/8nprr6z6c2.1).
