# Supplementary material for: Consumption of Energy Drinks and Their Effects on Sleep Quality among Students at the Copperbelt University School of Medicine in Zambia
Source: Sleep Disord. 2019 Feb 3;2019:3434507. doi: 10.1155/2019/3434507 (PMC6377981; doi:10.1155/2019/3434507)
Supplement: Supplementary Material — The questionnaire that was used in this research is provided as well as the definition of terms and list of acronyms/abbreviations. [file 3434507.f1.pdf]

SUPPLEMENTARY MATERIALS

S1: Questionnaire

PART A: STUDENT DETAILS

Age: ____

Gender: Male ☐ Female ☐

Program: ____

Year of study: 2 ☐ 3 ☐

PART B: ENERGY DRINK CONSUMPTION

1. In an average month for this academic year, do you drink more than one energy drink per month?

   Yes ☐ No ☐

   If your answer to this question is No, do not attempt any of the other questions in part B, proceed to part C. if your answer is Yes, proceed to question 2.

2. In an average month, how many energy drinks do you take?

   1-4 ☐ 5-10 ☐ 11 or more ☐

3. In an average week, how many energy drinks do you take?

   1-3 ☐ 4-7 ☐ 8 or more ☐

4. In an average day, how many energy drinks do you take?

   1 ☐ 2 ☐ 3 ☐ 4 ☐ 5 ☐ More than 5 ☐

5. Which energy drink do you usually take? (e.g. dragon, red bull, monster etc.)

   ____

6. In what situation(s) do you usually take energy drinks?

	Tick below where it applies to you
(a) Insufficient sleep	

(b) Needing more energy	
(c) Studying for an exam	
(d) Completing a course project, assignment or report.	
(e) Drinking with alcohol while partying	
(f) Treating a hangover	
(g) Other (Specify: ______________________________ ____	

7. How many energy drinks do you drink at one time for the reason(s) given in question 6 above?

   1 ☐ 2 ☐ 3 ☐ 4 ☐ 5 ☐ More than 5 ☐

8. what is the average number of times per month in the current term that you consume energy drinks for the reason(s) given in question 6 above?

   1 ☐ 2 ☐ 3 ☐ 4 ☐ 5 ☐ More than 5 ☐

PART C: SLEEP QUALITY, BASED ON PITTSBURGH SLEEP QUALITY INDEX (PSQI)

Instructions: The following questions relate to your usual sleep habits during the past month only. Your answers should indicate the most accurate reply for the majority of days and nights in the past month. **Please answer all questions.**

1. During the past month, what time have you usually gone to bed at night? ___________________
2. During the past month, how long (in minutes) has it usually taken you to fall asleep each night? ______________
3. During the past month, what time have you usually gotten up in the morning? _______________

4. During the past month, how many hours of actual sleep did you get at night? (This may be different than the number of hours you spent in bed.) ____________________

5. During the past month, how often have you had trouble sleeping because you…	Not during the past month	Less than once a week	Once or twice a week	Three or more times a week
a. Cannot get to sleep within 30 minutes				
b. Wake up in the middle of the night or early morning				
c. Have to get up to use the bathroom				
d. Cannot breathe comfortably				
e. Cough or snore loudly				
f. Feel too cold				
g. Feel too hot				
h. Have bad dreams				
i. Have Pain				
j. Other reason(s), please describe: ______________________________ ______________________________				
6. During the past month, how often have you taken medicine to help you sleep (prescribed or "over the counter")?				
7. During the past month, how often have you had trouble staying awake while driving, eating meals, or engaging in social activity?				

	No problem at all	Only a slight problem	Somewhat of a problem	A very big problem
8. During the past month, how much of a problem has it been for you to keep up enough enthusiasm to get things done?				
	Very Good	Fairly Good	Fairly bad	Very bad
9. During the past month, how would you rate your sleep quality overall?				

S2: List of abbreviations/acronyms

APA – American Psychological Association

CBU – Copperbelt University

BDS – Bachelor of Dental Surgery

BSc CM – Bachelor of Clinical Medicine

MBChB – Bachelor of Medicine and Surgery

PSQI – Pittsburgh sleep quality index

SOM – School of Medicine

SPSS – Statistical Package for the Social Sciences

S3: Definition of terms

Energy drink – Any caffeinated beverage [19]. In this research, the term will not be used to refer to other caffeinated beverages, namely coffee, tea and soft drinks.

Sleep - Sleep is a state of mind and body which typically occurs every night for several hours in which there is reduced awareness, metabolism, and physical relaxation [40].

Pittsburgh sleep quality index (PSQI) – PSQI is a 19-question self-administered questionnaire used to assess sleep quality [31].

Sleep latency – This is the time it takes from lying down for the night until sleep onset [41].

Sleep efficiency – It refers to the percentage of total time in bed actually spent sleeping [41].

Sleep duration – This is the amount of time spent sleeping [1].

Sleep disturbance - This refers to the difficulties in initiating and/or maintaining of sleep [31].

Daytime dysfunction – This refers to a lack of enthusiasm to carry out day to day activities [31].
